# Topical Administration of Acylated Homoserine Lactone Improves Epithelialization of Cutaneous Wounds in Hyperglycaemic Rats

**DOI:** 10.1371/journal.pone.0158647

**Published:** 2016-07-12

**Authors:** Lijuan Huang, Takeo Minematsu, Aya Kitamura, Paes C. Quinetti, Gojiro Nakagami, Yuko Mugita, Makoto Oe, Hiroshi Noguchi, Taketoshi Mori, Hiromi Sanada

**Affiliations:** 1 Departments of Gerontological Nursing/Wound Care Management, Graduate School of Medicine, The University of Tokyo, Hongo, Tokyo, Japan; 2 Department of Advanced Nursing Technology, Graduate School of Medicine, The University of Tokyo, Hongo, Tokyo, Japan; 3 Department of Life Support Technology (Molten), Graduate School of Medicine, The University of Tokyo, Hongo, Tokyo, Japan; NYU Langone Medical Center, UNITED STATES

## Abstract

Clinicians often experience delayed epithelialization in diabetic patients, for which a high glucose condition is one of the causes. However, the mechanisms underlying delayed wound closure have not been fully elucidated, and effective treatments to enhance epithelialization in patients with hyperglycaemia have not been established. Here we propose a new reagent, acylated homoserine lactone (AHL), to improve the delayed epithelialization due to the disordered formation of a basement membrane of epidermis in hyperglycaemic rats. Acute hyperglycaemia was induced by streptozotocin injection in this experiment. Full thickness wounds were created on the flanks of hyperglycaemic or control rats. Histochemical and immunohistochemical analyses were performed to identify hyperglycaemia-specific abnormalities in epidermal regeneration by comparison between groups. We then examined the effects of AHL on delayed epithelialization in hyperglycaemic rats. Histological analysis showed the significantly shorter epithelializing tissue (P < 0.05), abnormal structure of basement membrane (fragmentation and immaturity), and hypo- and hyperproliferation of basal keratinocytes in hyperglycaemic rats. Treating the wound with AHL resulted in the decreased abnormalities of basement membrane, normal distribution of proliferating epidermal keratinocytes, and significantly promoted epithelialization (P < 0.05) in hyperglycemic rats, suggesting the improving effects of AHL on abnormal epithelialization due to hyperglycemia.

## Introduction

Delayed wound healing is one of the most important complications of diabetes mellitus. The prolonged healing period of chronic ulcers substantially increases the risk of wound infection [1] and medical costs [2], and decreases the quality of life of diabetic patients [3].

Several approaches, including negative-pressure wound treatment and hyperbaric oxygen therapy, have been introduced for the treatment of chronic wounds [4, 5]. However, clinicians are increasingly experiencing diabetic patients with chronic wounds with little or no advance in wound closure or tylosis of the wound edge, despite using these approaches, suggesting that epithelialization is disrupted in diabetic patients [6]. Although diabetic patients have been reported to have abnormal inflammation, angiogenesis and granulation compared with non-diabetic patients [7–9], the underlying causes of delayed epithelialization in diabetes are poorly understood.

Epithelialization is initiated by the migration of basal keratinocytes over the provisional wound matrix. Then, a subset of keratinocytes adjacent to the wound bed undergoes mitosis, stratification and differentiation to restore the functionality of the epidermis [10]. Previous reports have revealed that high glucose conditions inhibit the migration and proliferation of normal human keratinocytes *in vitro*, suggesting that a high glucose condition is one of reasons for delayed wound closure in diabetes [11].

During epithelialization, the formation of an intact basement membrane between the regenerated epidermis and the granulation tissue is essential to re-establish the integrity and function of the skin. The main matrix components of the basement membrane include laminin 5 (LM5), type IV collagen (Col4) and fibronectin (FN) [12–15]. In particular, LM5 regulates the migration and proliferation of keratinocytes, and also provides a scaffold [16]. Abnormalities of LM5 expression in the basement membrane were previously reported to affect epithelialization in diabetic subjects [17]. These results suggest that disordered LM5 expression is a trigger of delayed epithelialization and that LM5 represents a therapeutic target for enhancing wound closure in hyperglycaemic patients.

In the present study, we focused on acylated homoserine lactone (AHL) as a candidate reagent that can enhance epithelialization in patients with hyperglycaemia. One member of the AHL family, N-(3-oxododecanoyl)-L-homoserine lactone, is a signalling molecule (autoinducer) involved in quorum sensing underlying the regulation of expression of several virulence genes dependent on the surrounding bacterial density in *Pseudomonas aeruginosa* [18]. Recently, some researchers have focused on the regulation of gene expression in mammalian host cells by bacterial AHL, a process termed “inter-kingdom signalling” [19]. We previously demonstrated the existence of AHL signalling in rat dermal fibroblasts [20]. Although the AHL signalling pathway in mammalian cells has not been fully elucidated, it can bind to PPARγ, which represents one possible mechanism by which AHL acts [21, 22]. PPARγ is abundantly expressed in keratinocytes [23] and its activation was found to increase LM5 expression [24]. Taken together, these earlier findings prompted us to hypothesize that topical AHL administration might enhance LM5 expression, and thereby reduce the delay in epithelialization caused by hyperglycaemia. Recently, Paes et al. [25] demonstrated the enhanced migration of the keratinocyte by the AHL administration in an in vitro scratch wound healing assay. They also reported the complete inhibition of the enhancing effect of AHL on the keratinocyte migration by the administration of the inhibitor for activator protein 1, of which interaction with Smad4 is essential for positive regulation of LM5 expression [26].

The present study was conducted primarily to examine the relationships between abnormalities of LM5 expression in the basement membrane and delayed epithelialization of cutaneous wounds in hyperglycaemic rats. We also attempted to develop a novel wound treatment consisting of topical AHL administration to correct the basement membrane abnormalities and promote epithelialization in diabetic rats.

## Materials and Methods

### Animals

Seven-week-old male Sprague-Dawley rats (SLC Japan, Shizuoka, Japan) were individually maintained in a specific pathogen-free room (temperature: 23 ± 2°C, humidity: 55 ± 10%). After acclimatization for 1 week, acute hyperglycaemia was induced by a single intraperitoneal injection of streptozotocin (55–60 mg/kg) dissolved in 0.1 M citrate buffer (pH 4.5). Rats with blood glucose levels > 300 mg/dL 1 week after injection and remaining at this level throughout the experimental period were used as hyperglycaemic model animals within 4 weeks after injection. Degeneration of nerve fibres and microangiopathy begin to appear 4–6 weeks after streptozotocin injection [27, 28]. Control rats were injected with citrate buffer alone.

The animal experiments were carried out in accordance with the Guidelines for Animal Experimentation published by the Japanese Association for Laboratory Animal Science (1987), and were approved by the Animal Research Committee of The University of Tokyo. Humane endpoints were the rapid weight loss of greater than 20% within a few days, laboured respiration accompanied by a strong abdominal breathing, and loosed skin elasticity suggesting dehydration. Researchers daily observed animal behaviour, and measured body weight. Any animals did not show the abnormal behaviour, although their body weight was slightly decreased after streptozotocin injection and wound creation.

### Wounding and AHL administration

In experiment 1, full-thickness round wounds (2-cm diameter) were created on the right flanks of hyperglycaemic and control rats using sterile scissors, under anaesthesia. The wounds were covered with polyurethane film dressing (3M, Saint Paul, MN, USA), which was changed daily until the end of the experiment. At each dressing change, the appearance of the wounds was photographed, and the wound area was measured using ImageJ software (National Institutes for Health, Bethesda, MD, USA), as described in our previous study [29].

In experiment 2, N-(3-oxododecanoyl)-L-homoserine lactone (Sigma, St. Louis, MO, USA) was dissolved at 10 mM with dimethyl sulfoxide (DMSO) and stored at –20°C as a stock solution. Just before use, stock solution was diluted to 10 μM with normal saline. As a control treatment, vehicle solution (0.1% DMSO diluted with normal saline) was applied for the contralateral wound.

As described in our previous report [20], two full-thickness wounds were created on both flanks of hyperglycaemic rats, one wound was treated with 10 μM N-(3-oxododecanoyl)-L-homoserine lactone (AHL group) and the other was treated with vehicle solution (vehicle group) for 24 hours on post-wounding day (PWD) 4.

In both experiments, five rats were euthanatized by overdose aspiration of isoflurane on PWD 7 and tissue samples including the wound site and surrounding skin were harvested for histological analysis. The other animals from each group were observed until complete wound closure.

### Histological and immunohistochemical analysis

The entire tissue harvested was fixed, embedded in paraffin, and cut in a sagittal direction into 4-μm-thick serial sections. Every 20th section was stained with haematoxylin and eosin (HE). The thickness and length of the regenerating epidermis, and the width of wound were measured in sections taken from near the centre of the wound. The epithelialization was evaluated with the length of the regenerating epidermis divided with the diameter (distance between the wound edges) of wound. All of the stained sections were observed to evaluate histological abnormalities. Neighbouring sections were used for immunohistochemistry. Antigen retrieval was carried out by incubating sections with 1 mg/mL proteinase K (37°C, 5 min) for LM5 and FN, or autoclaving (121°C, 15 min) in citrate buffer (pH 6.0) for Ki67. Immunoreactivities of antibodies for LM5 (Thermo Fisher Scientific, Fremont, CA, USA; Dilution 1:500) and FN (Cosmo bio, Tokyo, Japan; dilution 1:1000) were detected using the avidin–biotin complex method (VectaStain ABC standard kit, Vector, Burlingame, CA, USA), while those for Col4 (rabbit polyclonal; Millipore, Billerica, MA; dilution 1:100) and Ki67 (mouse monoclonal; Dako, Glostrup, Denmark; dilution 1:100) were detected using a polymerized secondary antibody (Histofine; Nichirei, Tokyo, Japan). The sections were counterstained with haematoxylin. For control staining, the primary antibodies were replaced with bovine serum albumin.

### *In vitro* experiments

Foetal rat skin keratinocytes (JCRB005, Health Science Research Resources Bank, Osaka, Japan) were cultured in Dulbecco’s modified Eagle Medium (Nacalai Tesque, Kyoto, Japan) supplemented with 10% foetal bovine serum (BioWest, Nuaillé, France) at 37°C under 5% CO_2_. One day after subculture, the medium was replaced with one of three types of medium supplemented with 0.1% AHL stock solution (AHL group) or DMSO (vehicle group), or unsupplemented medium (control group). Six hours after treatment, the expression levels of basement component genes were analysed by RT-PCR using a High Capacity cDNA reverse transcription kit, AmpliTaq Gold PCR master mix (Applied Biosystems, Carlsbad, CA, USA) and the primer pairs shown in Table 1. *Actin beta* (*Actb*) was used as an internal control. The expression levels of target genes were normalized to the level of *Actb* expression.

**Table 1:** Primer sequences and product sizes.

Gene	Accession No	Primer sequence (5’ →3’)		Product size (bp)
		Forward	Reverse	
*Actb*	NM_031144.2	CCCGCGAGTACAACCTTCTT	CCACGATGGAGGGGAAGAC	168
*Lm5*	XM_215963.4	GCATCAAAGACATCAGCATCG	GGTTGAAGCCTGGACAGCAT	158
*Fn*	NM_019143.1	TGGGACTGTACCTGCATTGG	CCCCAGACACAAACACTCCA	155
*Col4*	XM_225043.5	GCTACGATGGTTGCAATGGA	CCGATATTTGTCACGGTCCTC	154
*Mmp2*	NM_031054.1	ACAGGACCCTGGAGCTTTGA	CTTGCAGATCTCGGGAGTGA	175
*Mmp9*	NM_031055.1	GCGCTGGGCTTAGATCATTC	TGGGACACATAGTGGGAGGA	199
*Mmp11*	NM_012980.1	CAGAACCCAACGAGTGGACA	CCGATATTTGTCACGGTCCTC	185

Proliferative activity of keratinocytes was analysed using the WST-1 cell proliferation reagent (Roche Diagnostic, Indianapolis, IN, USA) 24 hours after treatment.

### Statistics

Data are shown as means ± standard deviation. The significance of differences between two groups was determined using Student’s *t*-test. Differences among three groups were analysed by analysis of variance followed by Tukey’s multiple comparison test. *P* values < 0.05 were considered to represent statistical significance.

## Results

### Experiment 1: Abnormal epithelialization in hyperglycaemic rats

**Fig 1:**
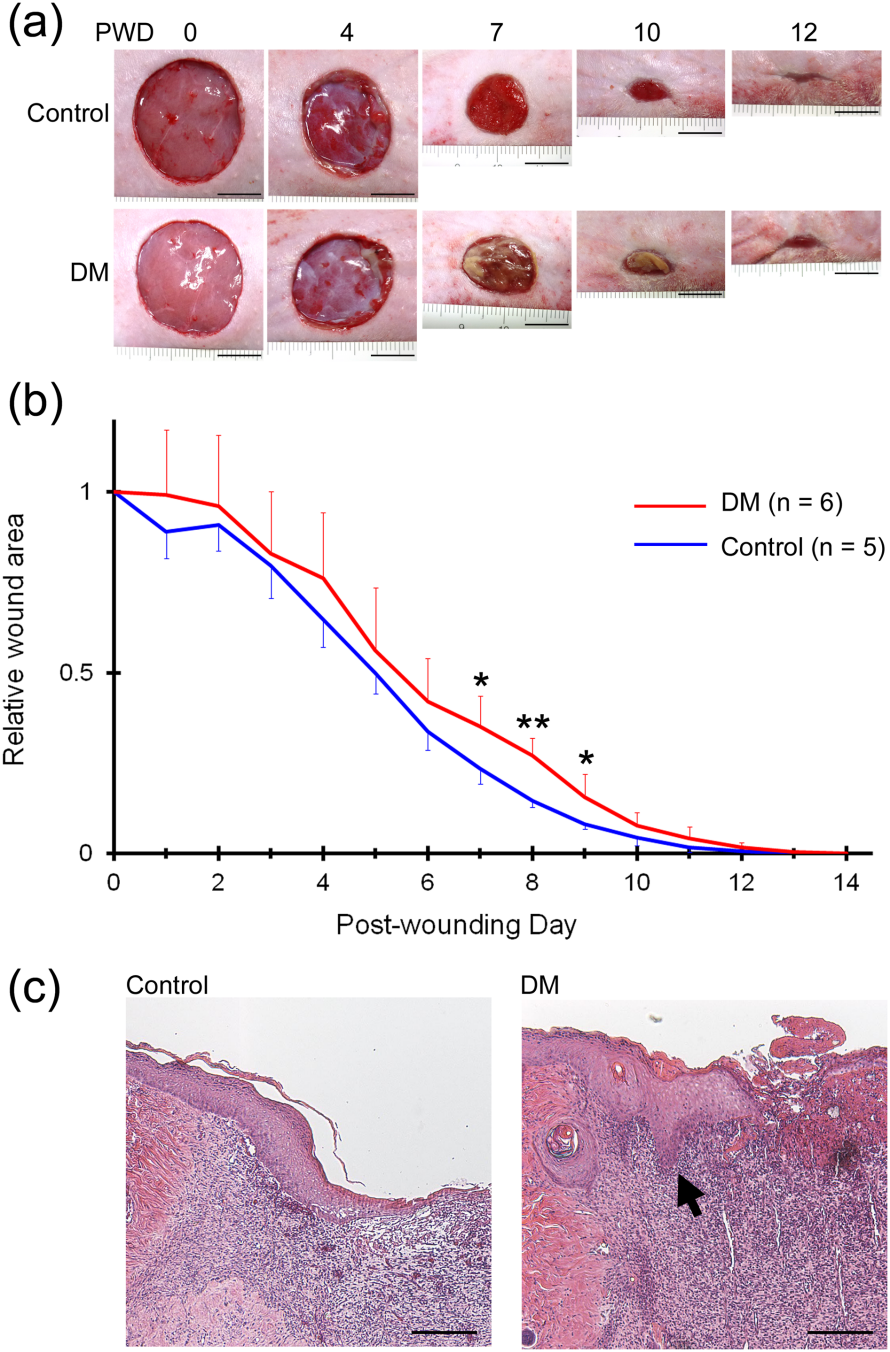
Wound healing and epithelialization in nondiabetic and diabetic rats in experiment 1. (a) Macroscopic observation of the healing of full-thickness wounds on the right flanks of nondiabetic (upper panels) and diabetic rats (lower panels) until complete wound closure. Delayed formation of granulation tissue on PWD 4, abundant necrotic tissue on the wound bed on PWD 7 and 10, and delayed epithelialization on PWD 7 were evident in diabetic rats. Scale bar = 1 cm. (b) The wound area measured on PWD 7–9 was significantly greater in diabetic rats (red line) than in nondiabetic rats (blue line). *P < 0.05; **P < 0.01. (c) HE staining showed that the regenerating epidermis was shorter and thicker in diabetic rats than in nondiabetic rats. The histological abnormalities, including invagination of regenerating epidermis into the granulation tissue (arrow), were specific to diabetic rats. Scale bar = 200 μm.

Full-thickness wounds were created on the right flanks of hyperglycaemic and control rats. The time to complete wound closure was 14 ± 0.4 days in hyperglycaemic rats, which was significantly longer than that in control rats (12 ± 0.5 days, *P* < 0.01). Macroscopic observation of the wounds indicated the presence of prolonged inflammation, as evidenced by redness and swelling of the wound edge, delayed formation of granulation tissue, and the presence of necrotic tissue on the wound bed in hyperglycaemic rats (Fig 1a). These results indicated that wound healing was delayed in hyperglycaemia. Epithelialization was observed along the entire wound edge on PWD 7 in control rats, but only along part of the wound edge in hyperglycaemic rats (Fig 1a). The wound area was significantly larger in hyperglycaemic rats than in control rats on PWD 7–9 (Fig 1b). Fig 1c shows the histology of sagittal sections taken near the centre of the wound. The diameters of wounds were not significantly different between two groups (9.11 ± 2.07 and 10.34 ± 1.79 mm in hyperglycaemic and control rats, respectively). The lengths of the regenerating epidermis divided with the diameters of wounds in these sections were 0.28 ± 0.085 and 0.14 ± 0.037 in hyperglycaemic and control rats, respectively, and these measurements were significantly different (*P* < 0.05). These results indicated that epithelialization was delayed under conditions of hyperglycaemia. Histological abnormalities specific to hyperglycaemic rats included invagination of the regenerating epidermis into the granulation tissue (Fig 1c). The thickness of the regenerating epidermis was also significantly greater in hyperglycaemic rats than in control rats (0.173 ± 0.0008 vs. 0.122 ± 0.0002 mm, *P* < 0.05), indicative of tylosis of the regenerating epidermis in the hyperglycaemic condition.

**Fig 2:**
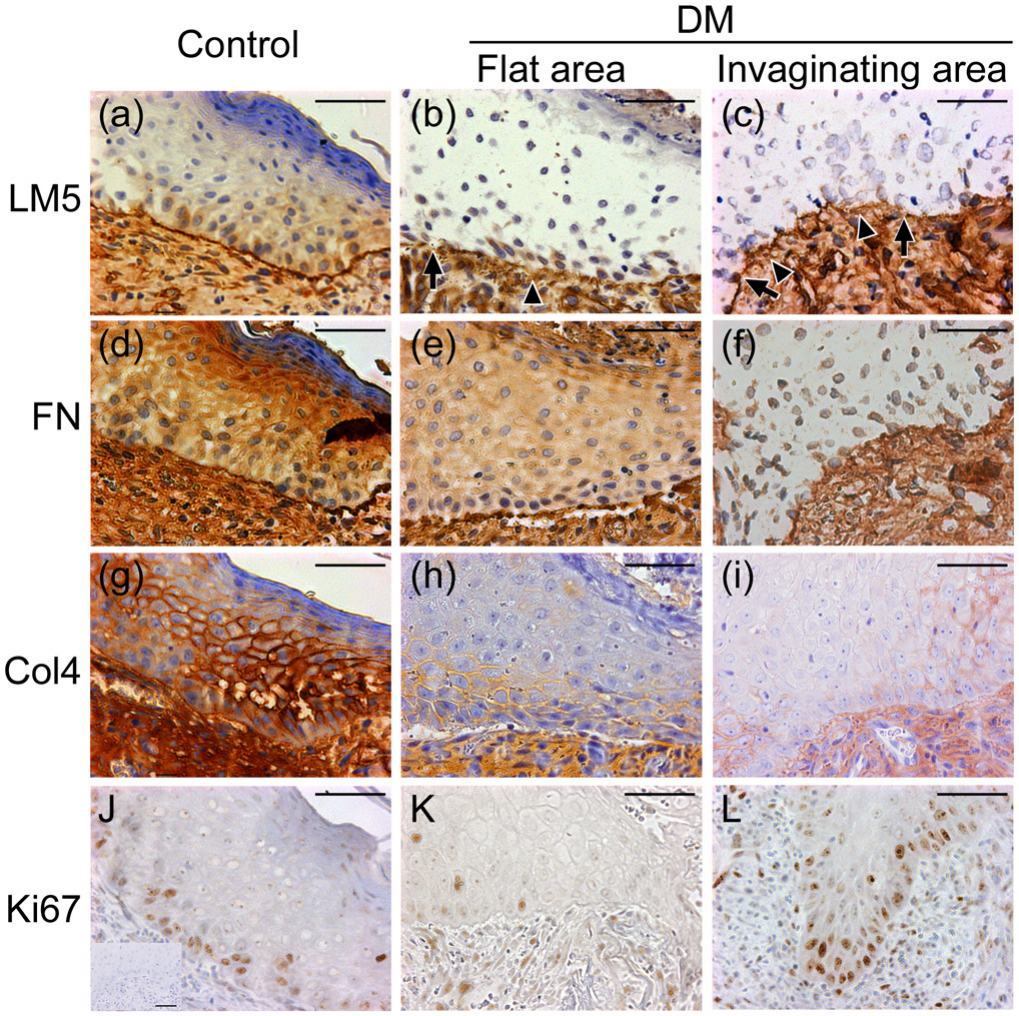
Immunohistochemistry of the basement membrane components LM5 (a–c), FN (d–f) and Col4 (g–i), and the proliferation marker Ki67 (j–l) in the regenerating epidermis of nondiabetic (a, d, g and j) and diabetic rats (b, e, h and k: flat area; c, f, i and l: invaginating area). In diabetic rats, immunoreactivity for basement membrane components was decreased compared with that in nondiabetic rats. Structural abnormalities of the basement membrane including fragmentation (arrows) and immaturity (arrowheads) were also observed. Scale bar = 200 μm.

The results of immunohistochemistry for basement membrane components are shown in Fig 2a–2i. Immunoreactivities for these components were remarkably decreased in the regenerating epidermis of hyperglycaemic rats compared with control rats. Observation of LM5-stained sections revealed hyperglycaemia-specific abnormalities of the basement membrane including fragmentation and immaturity, characterized by a wavy and thickened structure with decreased immunoreactivity. These abnormal structures were observed in all of the samples obtained from hyperglycaemic rats, but not in samples from control mice. Fragmentation of the basement membrane was mostly observed in the invaginating area of the regenerating epidermis.

Immunohistochemistry for Ki67 revealed a uniform distribution of proliferative keratinocytes in the basal layer of the regenerating epidermis in control rats (Fig 2j). In contrast, tissue samples from hyperglycaemic rats showed a patchy hyperproliferative area: Ki67-positive cells were mainly localized to the invaginating area, with a few within the flat area (Fig 2k and 2l).

### *In vitro* experiments

**Fig 3:**
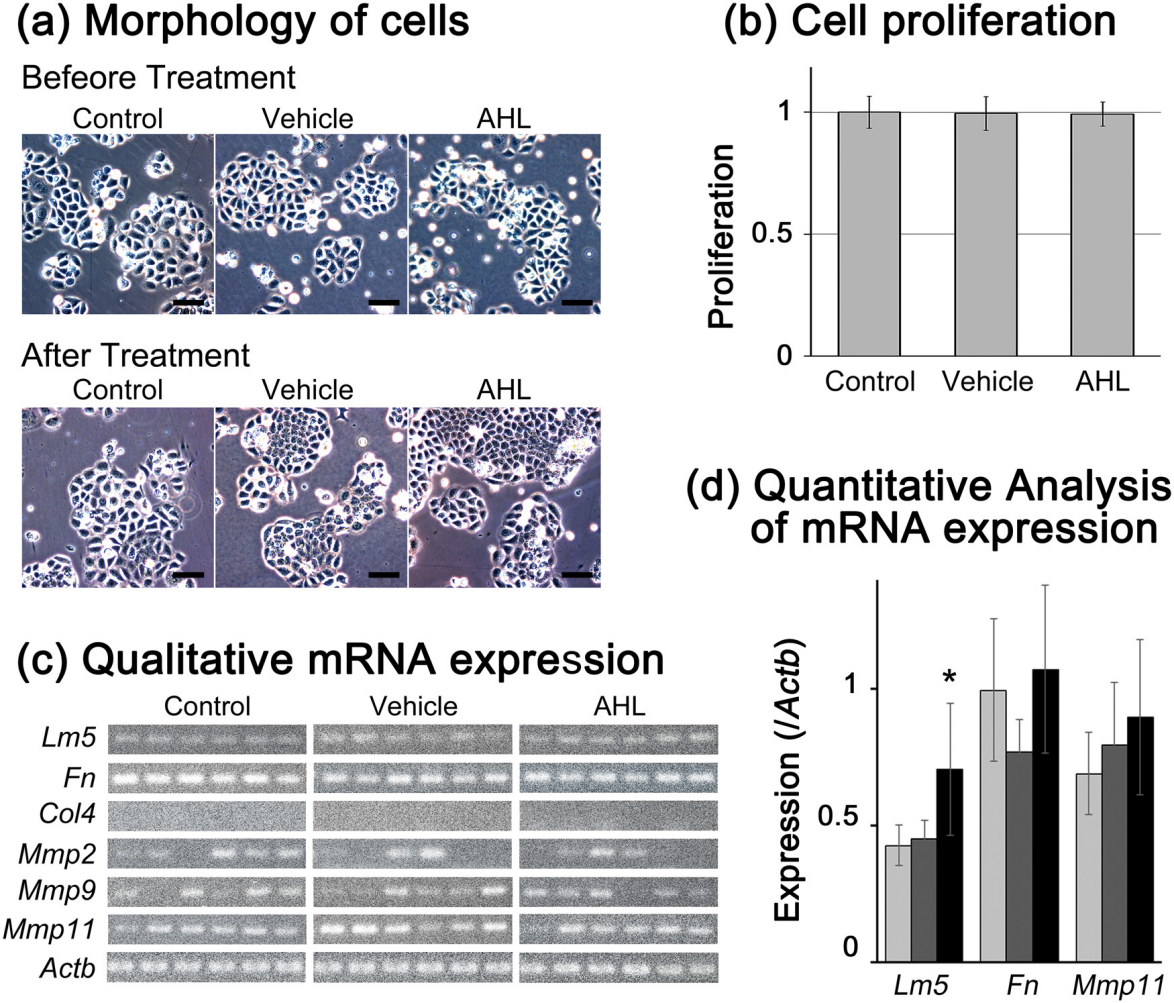
*In vitro* analysis of the effects of AHL on keratinocytes. Foetal rat skin keratinocytes were treated with 10 μM AHL, vehicle only (0.1% DMSO), or were untreated (control). (a) Six hours after treatment, there were no morphological changes in any group. Scale bar = 200 μm. (b) Twenty-four hours after treatment, there were no differences in cellular proliferation among the three groups. (c) Expression analysis of genes related to epidermal basement membrane. (d) Qualitative analysis revealed significant increases in *Lm5* expression in AHL-treated cells compared with the vehicle-treated and control groups. Light gray: Control, dark gray: Vehicle, and black: AHL. **P < 0.01.

First, we examined the effects of AHL on keratinocytes *in vitro*. AHL treatment did not affect the morphology of cells throughout the experimental period (Fig 3a). The keratinocytes showed a paving stone-like arrangement in all groups. The proliferative activities of cells were analysed 24 hours after treatment, and there were no differences among the three groups (Fig 3b). We determined the gene expression levels of basement membrane components including *Lm5*, *Fn*, *Col4*, *Mmp2*, *Mmp9* and *Mmp11* after 6 hours of treatment. The expression of *Col4* was not detected in any samples. The expressions of Mmp2 and Mmp9 were detected in part of samples, and the positivity was not different among groups. The intensity of expression was qualitatively compared in *Lm5*, *Fn* and *Mmp11*, since they were detected from all samples. Treatment with AHL significantly increased the expression of *Lm5* compared with the expression levels in control and vehicle groups (*P* < 0.01, Fig 3c). In contrast, the expression levels of *Fn* and *Col4* were not significantly different among the three groups.

### Experiment 2: AHL administration improves epithelialization in diabetic rats

**Fig 4:**
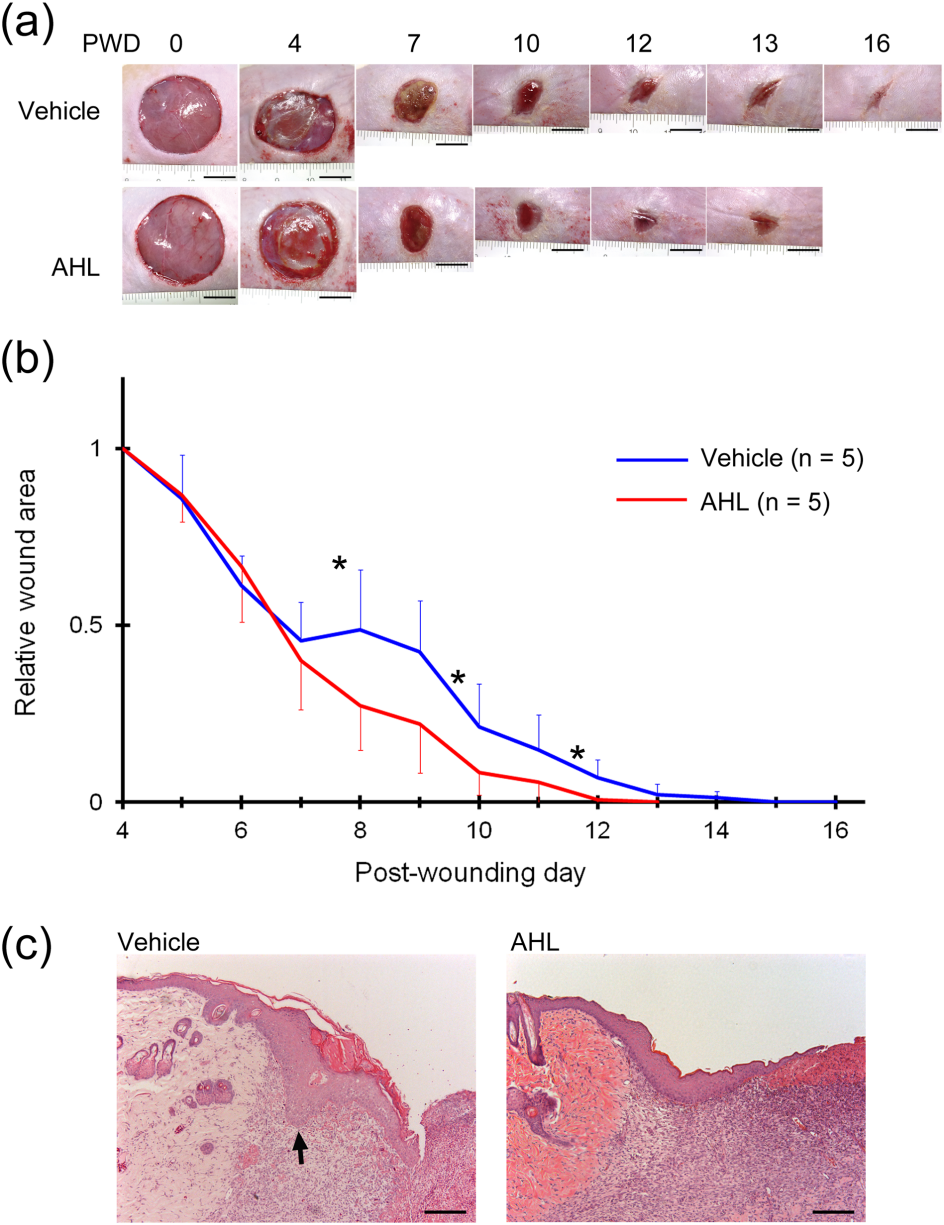
Effects of AHL on wound healing and epithelialization in diabetic rats in experiment 2. (a) Macroscopic observation of healing of full-thickness wounds on both flanks of diabetic rats. The wounds on the right and left flanks were treated with vehicle (upper panels) or AHL (lower panels) on PWD 4. The appearances of both wounds before treatment on PWD 4 were similar. In AHL-treated wounds, by PWD 7, the necrotic tissue had disappeared and regeneration of the epidermis was apparent. Scale bar = 1 cm. (b) The wound area of AHL-treated wounds (red line) on PWD 8, 10 and 12 was significantly smaller than that of vehicle-treated wounds (blue line). *P < 0.05. (c) HE staining showed improvements of the histological abnormalities, including tylosis and invagination (arrow), in AHL-treated wounds but not in vehicle-treated wounds. Scale bar = 200 μm.

Two full-thickness cutaneous wounds on the same hyperglycaemic rats were treated with AHL or vehicle solution for 24 hours on PWD 4. The wounds were morphologically similar before the treatment (Fig 4a). However, in AHL-treated wounds on PWD 7, the necrotic tissue disappeared and epidermal regeneration was apparent (Fig 4a). The wound area of AHL-treated wounds was significantly smaller than that of vehicle-treated wounds on PWD 8, 10 and 12 (all *P* < 0.05, Fig 4b). The mean time to wound closure was significantly shorter for AHL-treated wounds than for vehicle-treated wounds (12.7 ± 0.6 vs 15.0 ± 1.7 days, respectively, *P* < 0.05).

Because macroscopic observations showed significantly enhanced epithelialization from PWD 8, we histologically analysed the wound tissues on PWD 7 to determine the mechanisms underlying the effects of AHL on epithelialization. HE staining revealed no significant differences in the thickness of the regenerating epidermis and the diameter of the wound between the vehicle- and AHL-treated groups (thickness: 0.145 ± 0.0671 vs 0.132 ± 0.0531 mm, *P* = 0.491; diameter: 11.05 ± 3.24 vs 12.10 ± 7.23 mm, *P* = 0.796; in vehicle- and AHL-treated groups, respectively). However, it is worth noting that the length of regenerating epidermis divided with the diameter of wound in AHL-treated group (0.37 ± 0.148) was significantly longer than that in vehicle-treated group (0.26 ± 0.120, *P* = 0.014), and the invagination of the regenerating epidermis into granulation tissue was not observed in any of the tissue samples from AHL-treated wounds (Fig 4c).

**Fig 5:**
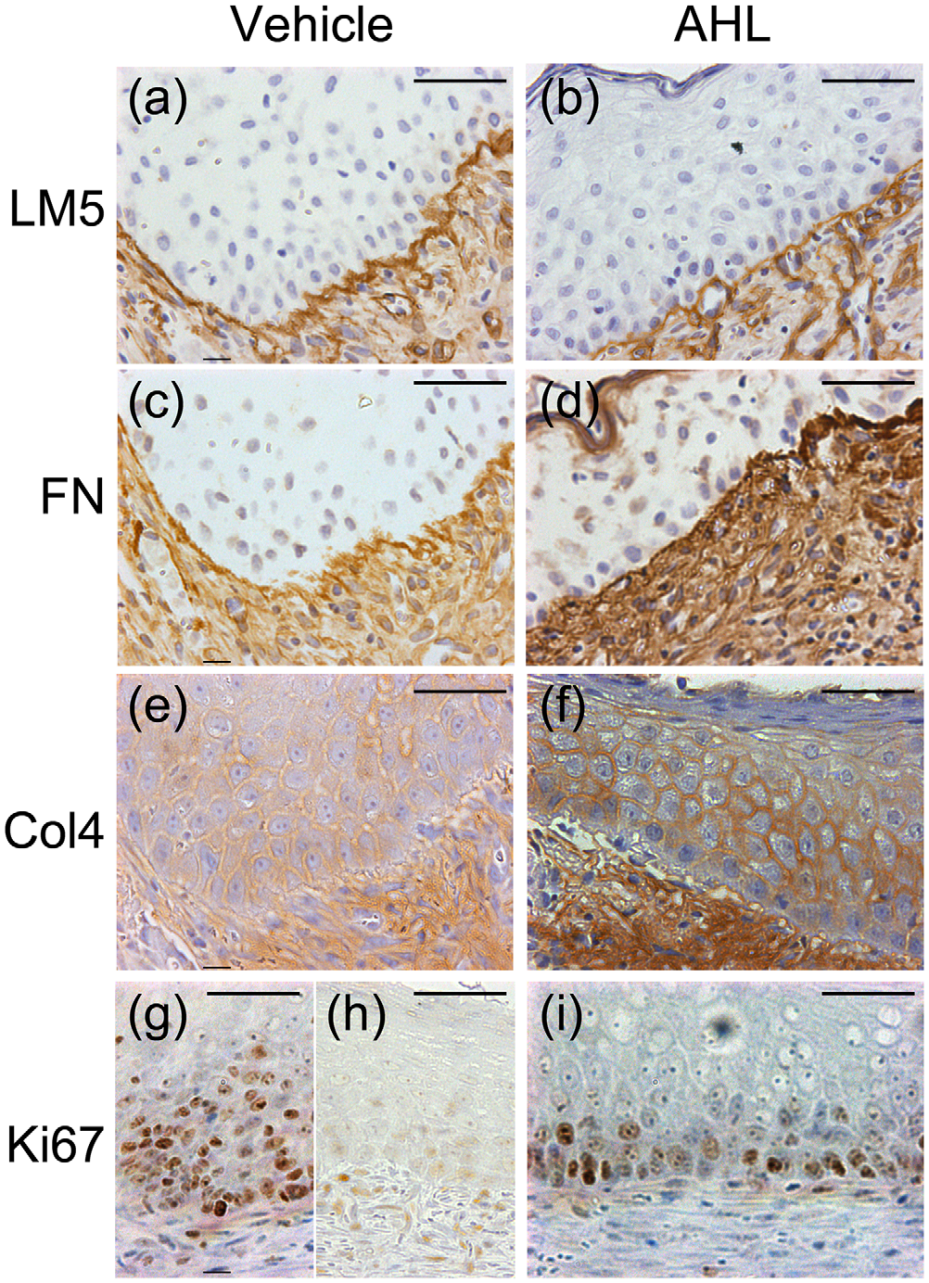
Immunohistochemistry for basement membrane components (a–f) and the proliferation marker Ki67 (g–i) in vehicle-treated (a, c, e and g) and AHL-treated wounds (b, d, f and i). Scale bar = 200 μm.

Immunohistochemistry for components of the basement membrane revealed increased Col4 immunoreactivity in the regenerating epidermis and obviously decreased structural abnormalities of the basement membrane, such as fragmentation and immaturity, which were both observed in two of six samples in the AHL-treated group, compared with five and six of six samples in the vehicle-treated group, respectively (Fig 5a–5f).

Proliferation studies revealed that most Ki67-positive cells were distributed in the invaginating area and showed hypoproliferation in the flat area of the regenerating epidermis in vehicle-treated wounds (Fig 5g and 5h). In comparison, Ki67-positive keratinocytes were uniformly distributed throughout the basal layer of the regenerating epidermis in AHL-treated wounds (Fig 5i).

## Discussion

In the present study, we found that topical administration of AHL enhanced epithelialization of a cutaneous wound in hyperglycaemic rats by restoring the structural abnormalities in the basement membrane.

In experiment 1, invagination of the regenerating epidermis into the granulation tissue, in addition to epidermal tylosis, was found to be a unique histological abnormality in hyperglycaemic rats, which exhibited delayed epithelialization. Although these characteristics have not yet been reported in diabetic patients or animals, we did see similar histological abnormalities in histological and/or immunohistochemical images of clinical samples published in previous reports [6], suggesting that this phenomenon is a common abnormality in the diabetic state.

The decreases in the levels of basement membrane components in the regenerating epidermis, which were consistent with those reported in several other studies [6, 15, 17], can be explained by the decreased mRNA expression of the genes encoding these components [30] and increased expression/activation of MMPs [31–33]. Immunohistochemistry for LM5 revealed structural abnormalities of the basement membrane including fragmentation and immaturity in hyperglycaemic rats. These structural abnormalities of the basement membrane were frequently observed in the invaginating area. Furthermore, the proliferating keratinocytes were stratified in the tip of the invaginating epidermis. The coincidence of invagination, structural abnormalities of the basement membrane and hyperproliferation suggests the existence of an association among these phenomena under the hyperglycaemic condition. Recently, it was reported that an LM5 fragment binds to the epidermal growth factor receptor and stimulates MMP-2 expression and cell migration as a possible mechanism leading to cancer invasion into the stroma [34]. The invagination of epithelializing tissue into granular tissue may result from the function of such LM5 fragments.

Hypoproliferation of epidermal keratinocytes is a major cause of delayed epithelialization in the hyperglycaemic rat. According to the previous reports [11], it is considered that high glucose directly inhibits keratinocyte proliferation. However, topical administration of AHL increased the numbers of proliferative keratinocytes in the regenerating epidermis of hyperglycaemic rats. Because AHL cannot directly enhance keratinocyte proliferation, the mechanisms underlying the enhancement of the epithelialization induced by AHL administration under conditions of hyperglycaemia likely involve other effectors. Basement membrane components and their receptors are known to function as regulators of proliferation, migration and differentiation of keratinocytes [35, 36]. Epithelialization in diabetes model animals can be enhanced by the topical administration of LM5 [17] and FN [37]. Moreover, in the present study, the structural abnormalities of the basement membrane were remarkably improved in AHL-treated wounds. These findings strongly suggested that the structural abnormalities of the basement membrane contributed to the delayed epithelialization and that preventing these abnormalities could improve epithelialization in the hyperglycaemic condition.

Our *in vitro* experiments indicated that AHL treatment significantly increased *Lm5* expression in keratinocytes. Although this effect might be a part of the mechanisms underlying the enhancement of epithelialization induced by AHL, the change in *Lm5* expression level is too small to fully explain the phenomenon. Because immunohistochemical analysis showed the improvement of structure of basement membrane, rather than the increase of expression of LM5, it is speculated that AHL might have an effect on the modification of LM5 and basement membrane structure. Further studies should be conducted to reveal the mechanisms of AHL effects on epithelialization completely.

To establish a novel therapeutic treatment using AHL for diabetic patients, possible interactions of factors such as insulin, leptin and ischemia with the effects of AHL should be investigated, because the pathophysiology of diabetes is complex, and many patients have severe complications [38]. Because higher concentration of AHL induces virulence expression in *P*. *aeruginosa* and apoptosis of mammalian cells [39], high doses of AHL should be avoided. Some studies have already attempted to determine the effective concentration range of AHL in mammalian cells *in vitro* [20]. Further studies are needed to determine the safety and effective dose range of AHL *in vivo*. In addition, the attempt to generalize these findings to human by the investigations in the better experimental animals, such as pig model, and the clinical diabetic patients.

Although, we focused only on N-(3-oxododecanoyl)-L-homoserine lactone in this study, the effects of several analogical molecules of AHL family, such as N-hexanoyl-L-homoserine lactone, N-decanoyl-L-homoserine lactone, N-dodecanoyl-L-homoserine lactone, have not investigated on wound healing. Futher investigations to compare their effects on wound healing are required for establishment the most effective therapeutic treatment of AHL in diabetic wounds.

In conclusion, we observed decreased expression of basement membrane components as well as structural abnormalities in the regenerating epidermis in hyperglycaemic rats. Structural abnormalities of the basement membrane led to disordered hypo- and hyperproliferation of basal keratinocytes, resulting in delayed epithelialization and histological abnormalities, particularly invagination. Treating the wound with AHL reversed some of these abnormalities in the basement membrane, particularly the abnormal keratinocyte proliferation and delayed epithelialization.
